# Implicit Trust between the Uyghur and the Han in Xinjiang, China

**DOI:** 10.1371/journal.pone.0071829

**Published:** 2013-08-19

**Authors:** Shen Zhang, Miao Xu, Xueting Li, Huizhen Fang, Shengmin Yang, Jia Liu

**Affiliations:** 1 Department of Psychology, University of Wisconsin-Whitewater, Whitewater, Wisconsin, United States of America; 2 State Key Laboratory of Cognitive Neuroscience and Learning, Beijing Normal University, Beijing, China; 3 School of Ethnology and Sociology, Minzu University of China, Beijing, China; Cuban Neuroscience Center, Cuba

## Abstract

Trust is a vital lubricant that increases the sense of security in social interactions. In this study, we investigated the intergroup trust between the Uyghur and the Han, the two largest ethnic groups in Xinjiang, China, with a Go/No-Go Association Task. Specifically, we instructed Uyghur and Han participants to respond to ethnic faces (Uyghur vs. Han) and trust/distrust words and measured the strength of the automatic associations between the faces and words for both in-group and out-group pairs. As expected, both ethnic groups showed implicit in-group trust and out-group distrust, but the Han group demonstrated stronger in-group trust and out-group distrust toward the Uyghur than the Uyghur group toward the Han. However, the magnitude of distrust of the Han toward the Uyghur was small to medium as compared with that reported by other intergroup relationship research. In addition, participant geographic location was associated with out-group distrust. These findings offer implications for developing effective strategies to encourage trust between conflicting groups.

## Introduction

Located on the northwest border of China and encompassing a sixth of the country’s territory, the Xinjiang Uyghur Autonomous Region is home to 47 ethnic groups. Among these groups, the Uyghur and the Han are the largest, comprising approximately 47% and 41% of the region’s population, respectively [1]. Recent years have seen violent incidents and ethnic clashes between these two groups, such as the Urumqi riot in July 2009, which resulted in the death of nearly 200 Han individuals. Previous studies on ethnic relations mainly investigate the role of long-term social, economic and political factors that underlie ethnic conflicts in this area (e.g., [2]–[4]). However, little is known about psychological factors underpinning the conflicts. We conducted the first study to directly examine the degree to which the Uyghur and Han trust each other using an implicit measure to inform the psychological underpinning of the ethnic conflict between the Uyghur and Han.

The Han in Xinjiang are immigrants from other provinces of China, having arrived over the previous hundreds of years [5]–[7]. They do not have a dominant religion but are mainly atheists, Buddhists and Daoists. The Uyghur are the offspring of the ancient nomad tribes in the Mongolian Prairie [8]–[10] and practice Islam. Besides factors such as physical appearance, religion and language that led to self-imposed segregation between the Uyghur and Han, changes from the planned economy to the market-oriented economy in recent years have led to fierce competition for resources and caused increasing gaps in wealth, which further exacerbate the ethnic tension (e.g., [2], [3], [11]–[15]).

Trust has been widely recognized as a vital social lubricant that can decrease the tension when interacting with others [16]. Trustworthiness is embedded in essential stereotype dimensions that people have toward out-groups, such as warmth (the Stereotype Content Model, [17]). Different levels of these perceived stereotypes or principles determine people’s approach-avoid tendencies [18], [19] and induce different emotions and behavioral tendencies toward those social out-groups [20]. For instance, a perceived lack of warmth arouses prejudice, negative behavior or even attacks [20]. Research directly examining trust has consistently shown that it leads to a reduction of explicit prejudice toward another cultural group (e.g., [21]), whereas distrust or biased anticipation leads to interaction avoidance between groups, such as between Whites and Blacks in the U.S. [22].

More recently, the literature on group conflicts highlights trust as a crucial element for facilitating positive intergroup relations [23], [24]. For example, out-group trust predicts positive emotions, such as intergroup forgiveness, among Protestants and Catholics in the Northern Ireland conflict [25]. Trust mediates the effect of empathy on Israeli Jews’ willingness to reconcile with Palestinians [26]. Importantly, on a behavioral level, trust mediates the effect of intergroup contact and facilitates agreements involving compromise and integrative solutions that benefit both groups, such as Catholics and Protestants in Northern Ireland [23] or Israelis and Palestinians [27]. Given the important role of trust in group conflict resolution and reconciliation [24]–[26], we explored intergroup trust between the Uyghur and Han in Xinjiang to reveal the psychological basis for their intergroup behaviors. In our study, trust was measured as the perceived or expected trustworthiness of another person (e.g., [28]), a relatively stable belief or attitude developed from social interactions.

Trust in the previous studies on the relationship between the Uyghur and Han is measured on solely an explicit level. However, explicit measures have several restrictions in comprehensively assessing the manifestation of trust. First, respondents may be unable to introspectively access their own attitudes [29], [30]. Second, Chinese social norms emphasize harmony and unity, so the Uyghur and Han may not be willing to reveal their true attitudes due to social desirability pressure (e.g., [29], [31]). In contrast, implicit measures reply on the automatic activation of attitude; therefore, individuals are usually unaware of their implicit attitudes [32]. In addition, implicit measures are especially suitable for sensitive issues related to social norms [33], [34]. Therefore, in this study, we focused on the degree to which the Uyghur and Han trusted each other on an implicit level and compared it with explicit level to gain a better understanding of this psychological variable along with an explicit measure of intergroup trust.

We assessed the Uyghur’s and Han’s implicit trust toward each other with a Go/No-Go Association Task (GNAT, [35]), which measures the strength of automatic cognitive associations between different categories and concepts. The GNAT is a variant of the IAT and has adequate to very good reliability [36], [37]. Specifically, Uyghur and Han participants were rapidly presented ethnic face images, either the Uyghur or Han, and two groups of attribute concepts, either trust or distrust words. In some trials, one pair of face stimuli and attribute words were the target (signal), such as Uyghur faces and trust words, and the other pair of group and attribute words were the distracter (noise), such as Han faces and distrust words. In other trials, the targets and distracters were reversed. Participants were required to categorize these stimuli quickly by pressing a single response key to target stimuli (e.g., Uyghur faces) and attributes (e.g., trust words) within a response deadline but doing nothing for the distracter (e.g., Han faces) and attributes (e.g., distrust words). Because for the pair of stimuli and evaluative attributes with a stronger association, participants show higher reaction accuracy than for the pair with a weaker association [35], the performance variability in differentiating specific targets (signal) from distracters (noise) is considered as an index of the implicit attitudes toward the targeted concept, measured as the sensitivity *d’* [35].

An advantage of using the GNAT to measure attitudes is that it can measure attitudes toward just one (social) group and does not need a second group as a reference to record participants’ relative attitudes toward the first group [35]. Therefore, the GNAT can separate “in-group trust” and “out-group trust,” both of which may constitute an ethnic trust bias. This distinction is particularly important because in-group attitude may interfere with the intergroup bias. According to the ingroup identity model [38], people in individualistic culture who consider themselves as separable individuals (e.g., American) have lower level of intergroup bias, than people in collectivistic culture (e.g., Han Chinese) [39]. Therefore, the examination of the amount of trust that participants have toward both their in-group and out-group offers a clear picture of intergroup attitudes.

In addition, we explored possible moderating effects of several important factors and participant demographics on the Uyghur’s and Han’s intergroup trust. For example, Xinjiang is divided into northern and southern regions by the Tianshan Mountains. The economic situation in the northern Xinjiang is significantly better than that in the southern Xinjiang [40]. We collected data on cities from both regions: Urumqi and Yining in northern Xinjiang and Korla in southern Xinjiang, to test whether the geographic location might moderate intergroup trust.

In sum, by combining psychological and ethnological measures, our study contributes to the field of intergroup research by providing data from an important multi-ethnic context that has rarely been explored before. Our study additionally offers implications for policies to promote intergroup harmony.

## Methods

### Participants

A sample of 448 individuals between 18 and 60 years of age from three cities in Xinjiang voluntarily participated in this study: 86 Uyghur and 68 Han in Korla, 72 Uyghur and 60 Han in Urumqi, and 74 Uyghur and 88 Han in Yining. A stratified random sampling technique was adopted, with ethnic group, gender and social-economic status as sampling strata. Participants were randomly recruited by the experimenter after public announcements and/or advertisements were made via local contacts. Each participant received 50 Chinese Yuan (about US $8) and a small gift as compensation for his or her time in the study. The study was approved by the institutional review board of Beijing Normal University, Beijing, China. Informed written consent was obtained from all participants before the study.

Several participants were excluded from analysis, including four who did not complete the GNAT task and five with low accuracy (at the chance level) or with extremely long average reaction latency (> Q3+3 IQR), resulting in 439 participants as the final sample for the data analysis. The sample comprised 215 Han, including 130 men and 84 women with 1 missing gender report. The average age of Han participants was 37.27 years (SD = 9.46). The sample also contained 224 Uyghur, including 132 men and 88 women with 4 missing gender reports, with an average age of 34.73 years (SD = 9.80). The two ethnic samples were roughly equivalent in terms of their social class, education background, and neighborhood ethnic composition (Table 1).

**Table 1:** Uyghur and Han participants’ demographic information.

	Uyghur	Han
**Social class**		
Farmer	21(9.38%)	24(11.16%)
Workers and retailers	63(28.13%)	75(34.88%)
Intellectuals class	63(28.13%)	72(33.49%)
Civil service	40(17.86%)	29(13.49%)
Imam	6(2.68%)	0(0%)
Unemployed	10(4.46%)	11(5.12%)
No record	21(9.38%)	4(1.86%)
**Educational background**		
Primary school and illiterate	14(6.25%)	7(3.26%)
Junior middle school	26(11.61%)	24(11.16%)
Senior middle school	33(14.73%)	57(26.51%)
College and university	97(43.30%)	108(50.23%)
No record	54(24.11%)	19(8.84%)
**Neighborhood ethnic composition**		
mostly in-group neighbors	80(35.71%)	123(57.21%)
mixed-group neighbors	40(17.86%)	43(20.00%)
mostly out-group neighbors	26(11.61%)	31(14.42%)
no record	78(34.82%)	18(8.37%)

*Note.* The intellectual class contains participants who are doctors, teachers and white-collar employees.

### Materials

Twenty facial pictures of Uyghur and Han Chinese (250×300 pixels), 10 for each ethnic group with 5 female and 5 male faces, were used as category stimuli for the GNAT. The number of face pictures was determined based on previous studies (e.g., [41]). The face pictures were taken from college students who were 18–25 years old. The pictures are easily categorized as either Uyghur or Han based on distinct facial features. All pictures were matched in their brightness and contrast. To further assess the attractiveness of face pictures, a group of Han and Uyghur college students were recruited to rate the attractiveness of each face picture on a 6-point Likert-type scale (1 = extremely unattractive, 6 = extremely attractive) in a pilot study. Besides that Uyghur faces were rated more attractive and that Uyghur participants tended to rate faces more attractive, there was a significant interaction of participant (Han vs. Uyghur) by type of face picture (Han faces versus Uyghur faces), showing that Uyghur participants rated faces from their own race more attractive than Han participants considered Han faces.

Two groups of words on trust and distrust from Burns, Mearns, & McGeorge’s study [42] were used as attribute stimuli for attitude measures in the GNAT, with 10 words in each group. Trust words included truthful, loyal, honor, confide, caring, reliable, be sure of, count on, dependable and honest. Distrust words included liar, deceitful, double-dealing, unreliable, devious, sly, dishonest, two faced, traitor and backstabber.

*Post-research questionnaire.* In addition to questions on participant age, gender, and other demographic information, the post-research questionnaire included an item measuring Uyghur/Han participants’ explicit out-group trust: “Do you think that most Han/Uyghur can be trusted?” This question was answered on a 5-point Likert scale from 1: “totally disagree” to 5: “totally agree.” Additional items investigated participants’ views of the ethnic relations between the Han and Uyghur and their attitude toward inter-ethnic marriages.

All materials were originally created in Chinese. A Uyghur version was created via translation and back-translation [43] by native Uyghur speakers to ensure the equivalence of materials, and therefore, the content was identical except for the language. Participants completed this study with materials in their language.

### Design and Procedure

The GNAT task was conducted under MATLAB 6.5 with psychophysics toolbox 2.54. Uyghur and Han participants took part individually in this experiment. They completed a GNAT task on a computer that was modified from Nosek and Banaji [35] in a 2 (Face Category: Uyghur, Han) × 2 (Word Attribute: Trust, Distrust) within-participant design.

Participants began with four 20-trial practice blocks of single face categories or word attributes in a random order to become familiar with these stimuli. Then, participants went through experimental blocks, each consisting of 80 critical trials (in addition to 10 practice trials), 40 with a single face stimulus (Han or Uyghur face) and 40 with a single word attribute (Trust or Distrust word), presented in a random order. Thus, the combination of face category and word attribute trials led to four experimental blocks in total (i.e., the four conditions): Han+Trust, Uyghur+Trust, Han+Distrust and Uyghur+Distrust, completed in a balanced order by each participant.

**Figure 1:**
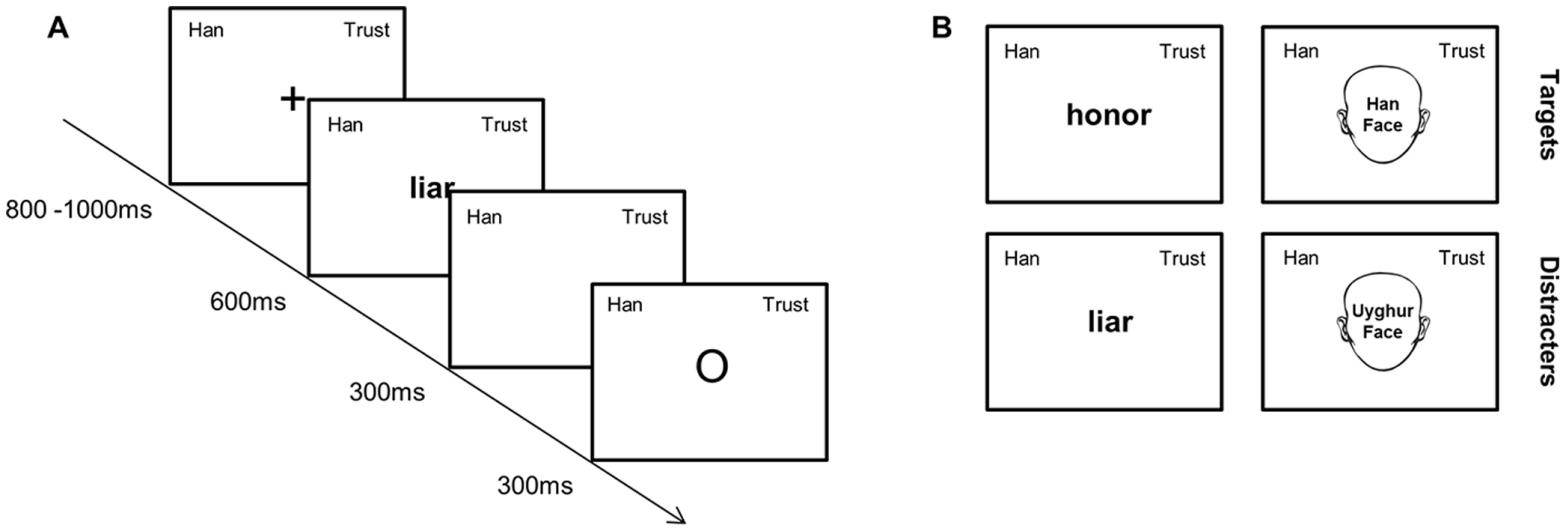
Stimuli and procedure. *A*. An example of a critical trial. *B.* Examples of word attributes (left row) and face stimuli (right row) in the *Trust+Uyghur* block. Word attributes included trust (upper) and distrust (lower) words, whereas face stimuli were Han faces (upper) and Uyghur faces (lower). Participants responded for targets (Trust words and Han faces), not for distracters (distrust words or Uyghur faces). To protect the privacy of the subjects of the photographs, the actual faces used in the study were not shown in the figure.

At the beginning of an experimental block, the target category (Uyghur or Han) and attribute (Trust word or Distrust word) appeared on the computer screen in the upper left and right quadrants respectively, as a reminder for participants and remained on the screen until the block was finished. At the same time, the appearance of a cross at the center of the computer screen signaled the start of each trial, and the cross was presented for 800–1000 ms. Then, the cross was replaced by a single stimulus item, either a face image or a trust/distrust word, presented for 600 ms. Participants were instructed to either (1) respond within 900 ms of the onset of the stimulus by pressing the space key as quickly as possible for stimuli belonging to the target category or attribute (a ‘go’ response, e.g., when the participant saw a Han face or a Trust word in the Han+Trust block), or (2) do nothing for stimuli that did not belong (a ‘no-go’ response, e.g., for a Uyghur face or a Distrust word in the same Han+Trust block). Then, either a green “O” (for participants’ correct ‘go’ or ‘no-go’ response) or a red “X” (for participants’ incorrect or no response within the 900-ms response deadline) appeared on the screen for 300 ms before the start of the next trial. Figure 1 shows an example of the sequential presentation of the stimuli in a trial and stimulus exemplars in a block. Participants’ responses in each trial and their reaction times from the “go” responses were recorded.

After the GNAT task, participants completed the post-research questionnaire.

### Data Analysis

Most measures of implicit attitudes are based on the association between the stimuli and the evaluative attributes in one’s mind [35], [44]. In our study, participants’ implicit attitudes of trust and distrust toward each ethnic group were reflected by the strength of participants’ automatic associations between the target category-Uyghur/Han faces and the trust/distrust attribute in the critical trials of the GNAT task. This association was measured by the sensitivity index *d’*, defined as a participant’s ability to make correct (‘go’ and ‘no go’) responses and distinguish instances of target category/attribute (e.g., Uyghur faces in a Uyghur+Trust block) from distracters (e.g., Han faces in the above block). Larger *d’* values indicated a stronger ability by participants to make such distinctions, and thus a stronger association by participants between the targeted category and attribute (e.g., Uyghur and trust in this example). We calculated this index based on participants’ *z*-transformed hits and false alarm rates in each of the four critical GNAT blocks according to the procedure outlined by Nosek and Banaji [35].

Given differences between the Han and Uyghur language (e.g., different word length) and that the Uyghur participants were relatively less experienced than the Han participants with verbal computer tasks, in this study we report *d’* results discriminating targets (faces and attributes) from distracters in each experimental block based only on participants’ responses to targeted face images but not word attributes. Data from five participants were excluded from analysis because they had at least one block of *d’* below zero [35].

## Results

### Implicit Trust

The Uyghur and Han participants’ automatic associations, the *d’* values, between their in-group or out-group ethnic face category and the trust/distrust words are shown in Table 2.

**Table 2:** Participants’ sensitivity (*d’*) in each ethnic group for each condition.

		Ingroup		Outgroup		
		Trust	Distrust	Trust	Distrust	n
**Uyghur**	Mean	3.83	3.14	3.32	3.75	224
	SD	0.94	0.64	1.06	1.00	
**Han**	Mean	3.87	2.94	3.13	3.76	210
	SD	1.05	1.15	1.13	1.01	

First, we examined whether we should assess in-group and out-group trust separately. To quantify the degree of the participants’ implicit trust toward their in-group and ethnic out-group, we calculated the *d’* difference scores by subtracting *d’* values for the incompatible block (e.g., in-group+distrust) from the *d’* values for the compatible block (e.g., in-group+trust) as an index for the strength of that attitude (e.g., the in-group trust), so that higher scores represent stronger implicit attitudes. Based on this index, participants’ implicit in-group and out-group attitudes of trust were only weakly correlated, *r* = −.083, *p* = .08, suggesting that these two components of trust measured by the GNAT were relatively independent. In further analyses, we therefore separately examined participants’ in-group and out-group trust attitudes.

To compare the trust of the Uyghur and Han toward their in-group and out-group, we conducted a mixed analysis of variance (ANOVA) on the *d’*, with targeted Face (In-group vs. Out-group) and Word (Trust vs. Distrust) as within-participant factors and participants’ Ethnicity (Uyghur vs. Han) as the between-participant factors. The main effect of Word attributes was significant, and the *d’* of trust trials (M = 3.54) was larger than that of distrust trials (*M = *3.40), *F* (1, 432) = 14.30, *p*<.001, *η^2^* = .03.

**Figure 2:**
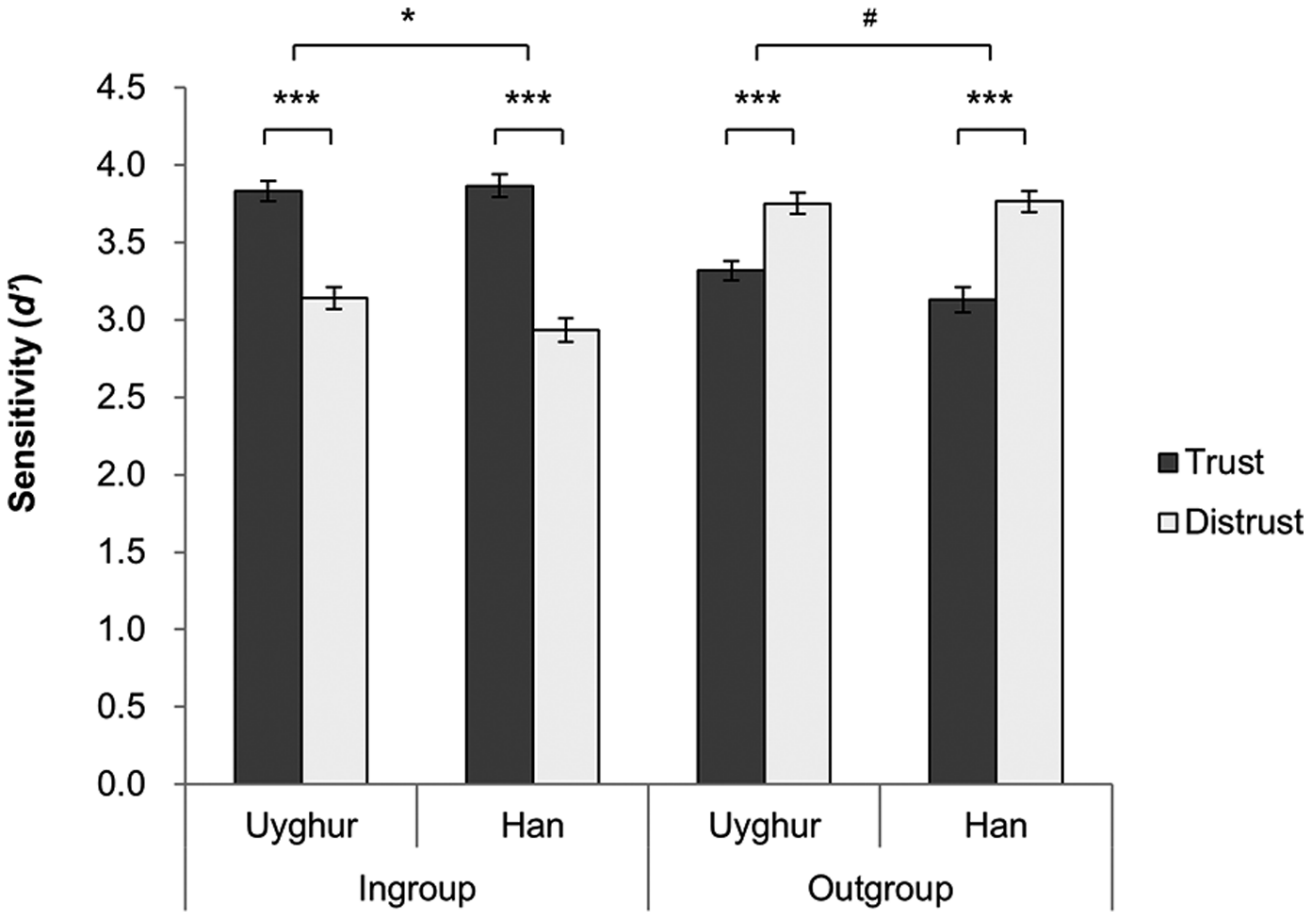
In-group trust and out-group distrust. *d’* values for Han and Uyghur participants toward *In−/out-group+Trust/Distrust*. Error bars denote standard error of mean (S.E.M.). ^*^ indicates p<.05, ^***^ indicates p<.001, ^#^ indicates p<.10.

Importantly, there was a significant Face × Word interaction effect (*F*(1, 432) = 288.80, *p*<.001, *η^2^* = .40), which was qualified by a significant Face × Word × Ethnicity interaction, indicating different attitudes toward their own group and the out-group among between the Uyghur and Han participants, *F*(1, 432) = 7.68, *p* = .006, *η^2^* = .02. In dissecting this three-way interaction effect, we found that the Uyghur participants displayed in-group trust, as reflected by the larger *d’* values for in-group face+trust words (*M* = 3.83) than for in-group face+distrust words (from corresponding distracters)(*M* = 3.14), *F*(1, 432) = 90.65, *p*<.001, *η^2^* = .17. Likewise, the Han participants showed in-group trust, with larger *d’* for in-group face+trust words (*M* = 3.87) than for in-group face+distrust words (*M* = 2.94), *F*(1, 432) = 153.55, *p*<.001, *η^2^* = .26. However, this pattern was stronger (indicating stronger in-group trust) among the Han than the Uyghur: *F*(1, 432) = 5.20, *p* = .023, *η^2^* = .012. Alternately, both Uyghur and Han participants showed out-group distrust, as reflected by a larger *d’* for group face+distrust words (*M* = 3.75, 3.76) than for out-group face+trust words (*M* = 3.32, 3.13), *F*(1, 432) = 31.35, 62.82, *ps*<.001, *η^2^* = .07,.13. The out-group distrust effect of the Han was marginally stronger than that of Uyghur participants, *F*(1, 432) = 3.24, *p* = .073, *η^2^* = .01, see Figure 2. Note that the asymmetry in trust that the Han showed stronger in-group trust and out-group distrust than the Uyghur cannot be accounted for by the attractiveness of face picture used in the GNAT test, because as shown in the pilot study (See Methods) Uyghur rated faces from their own race more attractive than Han rated Han faces.

We also ran the ANOVAs on the d’ based on participants’ responses for each trial in each block and ran them again after excluding the Uyghur participants who explicitly reported not being able to read some attribute words (>10% of total sample) and found the same significant pattern for the Face × Word × Ethnicity effect.

### Testing Moderators

Additional ANOVAs were conducted to test potential moderators for participants’ implicit trust/distrust attitudes.

*Gender.* We conducted a 2 (Face: In-group, Out-group) × 2 (Word: Trust, Distrust) × 2 (Ethnicity: Uyghur, Han) × 2 (Gender: Men, Women) ANOVA to examine gender as a potential moderator. The results revealed the same Face × Word × Ethnicity effect found in the main model (*p* = .020), which was not moderated by gender, *p* = .145. Instead, there was a significant Face × Word × Gender effect: *F*(1, 425) = 6.91, *p = *.009, *η* ^2^ = .02. Further simple effect analyses showed that both women and men demonstrated in-group trust, as reflected by the larger *d’* toward *in-group face+trust* words than *in-group face+distrust words*: *M* = 4.09 v. 3.20, *F*(1, 425) = 114.09, *p*<.001, *η* ^2^ = .21 for women, and *M = *3.69 v. 2.93, *F*(1, 425) = 124.59, *p*<.001, *η* ^2^ = .23 for men. These in-group trust attitudes were not moderated by gender (*p* = .231). Meanwhile, both genders demonstrated out-group distrust, with a larger *d’* toward *out-group face+distrust* words than for *out-group face+trust words*: *M* = 4.02 v. 3.32, *F*(1, 425) = 64.13, *p*<.001, *η* ^2^ = .13 for women, and *M* = 3.58 v. 3.17, *F*(1, 425) = 32.47, *p*<.001, *η* ^2^ = .07 for men. However, this out-group distrust pattern was stronger for women than men, *F*(1, 425) = 6.71, *p* = .010, *η* ^2^ = .02.

*Geographic location.* We tested participants’ geographic location as a moderator in the 2 (Face: In-group, Out-group) × 2 (Word: Trust, Distrust) × 2 (Ethnicity: Uyghur, Han) × 2 (Location: North Xinjiang (Urumqi, Yining) and South Xinjiang (Korla)) ANOVA. The results again showed the significant Face × Word × Ethnicity interaction found in the main model (*p* = .01) was moderated by the geographic locations of Han and Uyghur participants, *F*(2, 428) = 4.35, *p* = .013, *η* ^2^ = .02. To understand this interaction, we examined participants’ in- and out-group trust separately and found that participants’ geographic location affected their out-group trust (*F*(2, 428) = 3.40, *p* = .034, *η* ^2^ = .02 for the Word × Ethnicity × Location interaction) but not their in-group trust (*p* = .244). Further simple effect analyses showed a significant out-group distrust for both ethnic groups in the northern part of Xinjiang (i.e., Urumqi and Yining), *p*s < = .014. In contrast, little out-group distrust was observed for either ethnic group in the southern part of Xinjiang (Korla), except that the *d’* in differentiating between *out-group faces+distrust* words and *out-group faces+trust words* was marginally significant for the Uyghur, *F*(1, 428) = 3.28, *p* = .071.

To compare these implicit out-group trust attitudes, we calculated the *d’* difference scores (Table 3). Higher scores represented more implicit distrust. Besides the aforementioned results, the one-way ANOVA on these difference scores showed the out-group distrust of the Uyghur in Korla were marginally weaker than both Han in Korla and Han in Yining, *p*s = .08, with a Tukey correction for multiple comparisons.

**Table 3:** Average *d’* values (SD), related statistics and difference scores for each condition, specifically on out-group attitudes.

Participants	Location		*d’* Outgroup+Trust		*d’* Outgroup+Distrust	*d’* Difference	*F*	*n*	*η* ^2^
**Uyghur**		**Korla**		3.30 (1.17)	3.53 (1.08)	−.24	3.28^#^	78	
		**Urumqi**		3.40 (1.06)	3.99 (0.89)	−.59	18.80***	72	.04
		**Yining**		3.26(0.93)	3.75(0.99)	−.49	13.30***	74	.03
**Han**		**Korla**		2.88 (1.07)	3.62 (1.05)	−.75	28.31***	68	.06
		**Urumqi**		3.53(1.02)	3.90(0.97)	−.37	6.08*	58	.01
		**Yining**		3.06(1.17)	3.78(0.998)	−.73	33.08***	84	.07

*Note.* * indicates p<.05, *** indicates p<.001, ^#^ indicates p<.10.

### Explicit Out-group Trust and it Relation with Implicit Out-group Trust

Approximately 35% of Uyghur participants and 8–10% of Han participants did not complete questionnaire items on out-group trust. Among those who did, both Uyghur (*M* = 3.53, *SD* = 1.09) and Han (*M* = 3.43, *SD* = 1.10) reported moderate explicit trust toward the out-group. Interestingly, the self-reported out-group trust was not correlated with the implicit measure obtained from the GNAT task, *r = *.02, *p* = .77, *n* = 338, suggesting that explicit and implicit out-group trust are dissociable. Consistent with this observation, explicit and implicit out-group trust was accounted for by different social-psychological variables. For example, participants’ explicit out-group trust did not differ on ethnicity, gender or geographic location that modulated implicit out-group trust. Instead, explicit out-group trust was significantly correlated with participants’ reports on other explicit measures, including support for interethnic marriages and positive evaluations of current intergroup relations (both *p*<.001), whereas implicit out-group trust did not show any correlation with these explicit measures.

## Discussion

We conducted the first study on implicit trust between the Uyghur and Han in Xinjiang, China, and found that both ethnic groups implicitly trusted their own group and did not trust the other (out-) group. These findings are in line with previous intergroup research on explicit trust and in-group favoritism; trust is extended to fellow in-group members but not to out-group members [45]–[49]. Importantly, the Han group demonstrated stronger in-group trust and out-group distrust toward the Uyghur than the Uyghur group toward the Han. In short, our study provides the first empirical evidence on the psychological underpinning of the ethnic conflict between the Uyghur and Han based on implicit measures, supplementary to previous studies based on explicit measures.

To gauge the amount of in-group trust and out-group distrust between the Uyghur and Han, we compared the *d’* values acquired in this study with those from other studies that used the GNAT to examine implicit intergroup attitudes. Nosek and Banaji [35] investigated attitudes toward Whites and Blacks in the U.S., and found that participants (>60% White) showed positive attitudes toward Whites, Cohen’s *d* = .77, and negative attitudes toward Blacks, an out-group to most participants (96%), Cohen’s *d* = .51. In a study conducted in Australia on non-Muslim Whites’ attitudes toward Muslims and non-Muslims [50], the Cohen’s *d* of participants’ negative attitude toward Muslims was 1.41 (converted from *t*(42) = 9.11, based on the formula 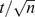. n: number of participants). In our study, the in-group trust had a Cohen’s *d* = .60 for the Han and.46 for the Uyghur participants, and the out-group distrust had a Cohen’s *d* = .38 for the Han and.27 for the Uyghur. These effects are in the small to medium range, similar to the levels of racial bias found in Nosek & Banji [35] and lower than the bias of Australian Non-Muslim Whites toward the Muslim out-group. The cross-study comparison suggests that ethnic tensions between the Uyghur and Han are not as intense as originally speculated [11], [51]–[54]. Alternatively, the nature of the relationship between a majority group and Islamic minorities is likely to vary based on different cultural contexts.

Although the Uyghur and Han showed both in-group trust and out-group distrust, asymmetric implicit trust between the Uyghur and Han was found. Han participants showed stronger in-group trust and out-group distrust than the Uyghur did toward the Han. The asymmetrical pattern is probably due to the time when the study was conducted. This study was conducted shortly after the Urumqi clashes in which the victims were mostly Han. The stronger distrust pattern might reflect the aftermath of this ethnic conflict on Han individuals. In line with this conjecture, Americans demonstrated high levels of discrimination against Arabs and Muslims after the 911 attacks in 2001 (e.g., [55], [56]). Similarly, majority ethnic groups in European countries reported biases against Middle Eastern immigrants or Muslims because of terrorist activities [57], [58], and participants in studies using the implicit attitude test (IAT) also demonstrated more negative implicit attitude associations toward Muslims than Blacks [59]. Various factors might influence the intergroup trust between the Uyghur and Han. However, in the present study, we only described the status of the intergroup trust after the ethnic conflict. In order to explain how the history of ethnic conflict affects the intergroup relationship, we will include more comparison groups and performance longitudinal studies in Xinjiang the further studies.

Interestingly, the geographic location plays a significant role for participants’ out-group trust attitudes for each ethnic group. Specifically, we found significant out-group distrust for both ethnic groups in the northern part of Xinjiang (i.e., Urumqi and Yining), whereas little out-group distrust for either group was observed in the southern part (Korla). One possibly interpretation is that the recent ethnic conflict occurred mainly in the northern cities, especially Urumqi. However, the differences between the north and south of Xinjiang result from many factors, not just recent incidents, cultural interactions or ethnic populations. Thus, the exact causes of the geographic differences are unclear and needed to be systematically studied in the future. Regardless, our current findings suggest that ethnic policies might need to be flexible and consider geological differences for effectively promoting positive intergroup relations.

Finally, gender moderated participants’ implicit trust. We found high levels of distrust toward the out-group among women, although women demonstrated similar levels of in-group trust as men. These findings complement previous research on gender and general/social trust. For example, women are possibly more afraid of exploitation and potential loss from mistrust due to their lack of social capital relative to men [60]. Thus, women in our study trusted strangers less than their male counterparts [61]. Our findings further showed that the group status of strangers, whether they are in-group or out-group members, matters at an implicit level.

The aforementioned findings from the implicit measure were not correlated with participants’ self-reported out-group trust. The disparity is in accordance with research on explicit and implicit social attitudes, which often fails to find correlations between these two types of measures [44], [62] (for a review, see [63]). Participants’ self-reported out-group trust in our study likely follows the mainstream Chinese social norms that emphasize harmonious intergroup relationships, especially considering the missing reports on those explicit attitude measures. In addition, the surprising distrust toward the Han, the self-reported support for interethnic marriage and the positive evaluations of the interethnic group relationships among the Uyghurs may have reflected the efforts of these individuals to follow social norms in their oral reports but not necessarily their genuine attitudes. These results indicated the importance of collecting multiple indexes, especially using indirect, subtle measures, to achieve a better understanding of inter-group attitudes and relationships.

One limitation of the current study is that we only sampled several urban areas in Xinjiang. While our motivation was to have comparable groups of Uyghur and Han participants, because the Han mostly dwell in the cities and the overall Han population has a higher percentage of professionals and intellectuals than the Uyghur, our matched Uyghur sample did not represent the overall background distribution of the Uyghur in Xinjiang. Therefore, one should be cautious when generalizing the obtained attitudes to the entire Uyghur population. Analyses based on socio-economic status should be pursued in the future for a more nuanced understanding of the ethnic relationships.

To summarize, we explored the intergroup trust between the Uyghur and Han as an important first step toward disentangling the psychological mechanisms of the ethnic conflict between the Uyghur and Han. We focused on implicit trust bias, and our study offers a more complete analysis of the ethnic attitudes between the Uyghur and Han in Xinjiang than past research that mainly relied on self-reports. Meanwhile, our study provides an interesting contrast to other interethnic relationships and suggests that the nature of ethnic relationships may depend on specific cultural contexts. In addition, our findings suggest scrutinizing specific areas in Xinjiang to adopt different strategies to promote intergroup trust and future directions for studying how trust/distrust between the Uyghur and Han translates into behavioral tendencies in ethnic conflicts.

## References

[1] Xinjiang Uygur Autonomous Region Bureau of Statistics (2010) Xinjiang Statistical Yearbook; Jin J, editor: China Statistics Press.

[2] Li J, Jiang L (2001) The relationship between Uygur and Han in Xinjiang: A Survey of Aydingkol Xiang in Turpan. In: Ma R, Pan N, Zhou X, editors. Studies on the Development of Ethnic Communities in China: Peking University Press.

[3] Li X (2012) On the Trend of Ethnic relations in Xinjiang and its Influencing Factors. Journal of the Second Northwest University for Nationalities 103: 40–48.

[4] Wang H, Yang S (2007) Investigation and Analysis of Inter-ethnic Relations in Kuche County,Xinjiang. North West Ethno-national Studies 53: 14–24.

[5] Li F (2010) Explore the historical significance of multi-ethnic pattern in Xinjiang. Xinjiang Chorography 2010(4): 46–49.

[6] Li J (2009) A Discussion on the Han Immigration in Xinjiang during the Period of Republic of China. China’s Borderland History and Geography Studies 19: 126–138.

[7] Wang Z (1997) The history of Han’s migration to Chian’s Northwest borer areas. Historical and Geographical Review of Northwest China 1997(1): 14–20.

[8] Liu N (2006) Primary research on the population of Uyghur. Research of China’s Frontier Archaeology 2006(5): 271–277.

[9] Wei L (1999) Rethinking the orgin, formation and development of Uyghur. Ethno-National Studies 1999(4): 83–88.

[10] Yang S (2008) History of Huihe: Guangxi Normal University Press.

[11] Han E, States U (2010) Boundaries, Discrimination, and Interethnic Conflict in Xinjiang, China. International Journal of Conflict and Violence 4: 244–256.

[12] Howell A, Fan C (2011) Migration and Inequality in Xinjiang: A Survey of Han and Uyghur Migrants in Urumqi. Eurasian Geography and Economics 52: 119–139.

[13] Ma R (1995) Sociology studies on Ethnical relation: Sociology of Ethnicity. Sociology of Ethnicity 2: 2–22.

[14] Ma R (2009) Several Issues in Ethnic Relations in Urban China. North West Ethno-national Studies 60: 6–19.

[15] Ma X (2011) Investigation Inter-ethnic Relations of Uyghur and Han in Hetian, Xinjiang. Social Sciences in Xinjiang 2011(6): 64–69.

[16] Kramer RM (1999) Trust and distrust in organizations: emerging perspectives, enduring questions. Annu Rev Psychol 50: 569–598. 15012464 10.1146/annurev.psych.50.1.569

[17] Fiske ST, Cuddy AJ, Glick P, Xu J (2002) A model of (often mixed) stereotype content: competence and warmth respectively follow from perceived status and competition. J Pers Soc Psychol 82: 878–902. 12051578

[18] Cacioppo JT, Gardner WL, Berntson GG (1997) Beyond bipolar conceptualizations and measures: the case of attitudes and evaluative space. Pers Soc Psychol Rev 1: 3–25. 15647126 10.1207/s15327957pspr0101_2

[19] Peeters G (2002) From good and bad to can and must: subjective necessity of acts associated with positively and negatively valued stimuli. Eur J Soc Psychol 32: 125–136.

[20] Cuddy AJC, Fiske ST, Glick P (2008) Warmth and competence as universal dimensions of social perception: The stereotype content model and the BIAS map. Adv Exp Soc Psychol 40: 61–149.

[21] Turner RN, Hewstone M, Voci A (2007) Reducing explicit and implicit outgroup prejudice via direct and extended contact: The mediating role of self-disclosure and intergroup anxiety. J Pers Soc Psychol 93: 369–388. 17723054 10.1037/0022-3514.93.3.369

[22] Shelton JN, Richeson JA (2005) Intergroup contact and pluralistic ignorance. J Pers Soc Psychol 88: 91–107. 15631577 10.1037/0022-3514.88.1.91

[23] Tam T, Hewstone M, Kenworthy J, Cairns E (2009) Intergroup trust in Northern Ireland. Pers Soc Psychol Bull 35: 45–59. 19106077 10.1177/0146167208325004

[24] Tropp LR (2009) The Role of Trust in Intergroup Contact: Its Significance and Implications for Improving Relations between Groups. Improving Intergroup Relations: Blackwell Publishing Ltd. 91–106.

[25] Noor M, Brown R, Gonzalez R, Manzi J, Lewis CA (2008) On positive psychological outcomes: what helps groups with a history of conflict to forgive and reconcile with each other? Pers Soc Psychol Bull 34: 819–832. 18388252 10.1177/0146167208315555

[26] Nadler A, Liviatan I (2006) Intergroup reconciliation: effects of adversary’s expressions of empathy, responsibility, and recipients’ trust. Pers Soc Psychol Bull 32: 459–470. 16513799 10.1177/0146167205276431

[27] Maoz I, Ellis DG (2008) Intergroup Communication as a Predictor of Jewish-Israeli Agreement With Integrative Solutions to the Israeli–Palestinian Conflict: The Mediating Effects of Out-Group Trust and Guilt. J Commun 58: 490–507.

[28] Rotter JB, Stein DK (1971) Public Attitudes Toward the Trustworthiness, Competence, and Altruism of Twenty Selected Occupations. J Appl Soc Psychol 1: 334–343.

[29] Nisbett RE, Wilson TD (1977) Telling more than we can know: Verbal reports on mental processes. Psychol Rev 84: 231–259.

[30] Wilson TD, Lindsey S (2000) Schooler TY (2000) A model of dual attitudes. Psychol Rev 107: 101–126. 10687404 10.1037/0033-295x.107.1.101

[31] Dunton BC, Fazio RH (1997) An Individual Difference Measure of Motivation to Control Prejudiced Reactions. Pers Soc Psychol Bull 23: 316–326.

[32] Greenwald AG, Banaji MR (1995) Implicit social cognition: attitudes, self-esteem, and stereotypes. Psychol Rev 102: 4–27. 7878162 10.1037/0033-295x.102.1.4

[33] Kraus SJ (1995) Attitudes and the Prediction of Behavior: A Meta-Analysis of the Empirical Literature. Pers Soc Psychol Bull 21: 58–75.

[34] Towles-Schwen T, Fazio RH (2006) Automatically activated racial attitudes as predictors of the success of interracial roommate relationships. J Exp Soc Psychol 42: 698–705.

[35] Nosek BA, Banaji MR (2001) The Go/No-go Association Task. Social Cognition 19: 625–666.

[36] Burns C, Conchie S (2011) Measuring implicit trust and automatic attitude activation. In: Lyon F, Mollering G, Saunders M, Hatzakis T, editors. Handbook of Research Methods on Trust. London: Edward Elgar.

[37] Williams BJ, Kaufmann LM (2012) Reliability of the Go/No Go Association Task. J Exp Soc Psychol 48: 879–891.

[38] Dovidio JF, Saguy T, Shnabel N (2009) Cooperation and Conflict within Groups: Bridging Intragroup and Intergroup Processes. J Soc Issues 65: 429–449.

[39] Triandis HC, McCusker C, Hui CH (1990) Multimethod probes of individualism and collectivism. J Pers Soc Psychol 59: 1006–1020.

[40] Li Y, Ren F (2012) The warning of economic differences between southern and northern Xinjiang. Journal of Arid Land Resources and Environment 26: 1–7.

[41] Williams BJ, Kaufmann LM (2012) Reliability of the Go/No Go Association Task. J Exp Soc Psychol 48: 879–891.

[42] Burns C, Mearns K, McGeorge P (2006) Explicit and implicit trust within safety culture. Risk Anal 26: 1139–1150. 17054521 10.1111/j.1539-6924.2006.00821.x

[43] Brislin RW (1970) Back-Translation for Cross-Cultural Research. J Cross Cult Psychol 1: 185–216.

[44] Greenwald AG, McGhee DE, Schwartz JL (1998) Measuring individual differences in implicit cognition: the implicit association test. J Pers Soc Psychol 74: 1464–1480. 9654756 10.1037//0022-3514.74.6.1464

[45] Allport GW (1954) The nature of prejudice. Cambridge, Mass.,: Addison-Wesley Pub. Co. 537 p. p.

[46] Brewer MB (1979) In-group bias in the minimal intergroup situation: A cognitive-motivational analysis. Psychol Bull 86: 307–324.

[47] Insko CA, Schopler J, Hoyle RH, Dardis GJ, Graetz KA (1990) Individual-group discontinuity as a function of fear and greed. J Pers Soc Psychol 58: 68–79.

[48] Otten S, Moskowitz GB (2000) Evidence for Implicit Evaluative In-Group Bias: Affect-Biased Spontaneous Trait Inference in a Minimal Group Paradigm. J Exp Soc Psychol 36: 77–89.

[49] Tajfel H (1982) Social psychology of intergroup relations. Annu Rev Psychol 33: 1–39.

[50] Gonsalkorale K, Hippel Wv, Sherman JW, Klauer KC (2009) Bias and regulation of bias in intergroup interactions: Implicit attitudes toward Muslims and interaction quality. J Exp Soc Psychol 45: 161–166.

[51] Bovingdon G (2010) The Uyghurs: Strangers in Their Own Land: Columbia University Press.

[52] Kaltman B (2007) Under the Heel of the Dragon: Islam, Racism, Crime, and the Uighur in China: Ohio University Press.

[53] Yee H (2003) Ethnic Relations in Xinjiang: A survey of Uygur-Han relations in Urumqi. Journal of Contemporary China 12: 431–452.

[54] Yee H (2005) Ethnic Consciousness and Identity: A Research Report on Uygur-Han Relations in Xinjiang. Asian Ethnicity 6: 35–50.

[55] Cainkar L (2002) No longer invisible: Arab and Muslim exclusion after September 11. Middle East Report 224: 22–29.

[56] Coryn CL, Beale JM, Myers KM (2004) Response to September 11: Anxiety, Patriotism, and Prejudice in the Aftermath of Terror. Current Research in Social Psychology 9: No Pagination Specified.

[57] Allen C, Nielsen SJ (2003) Islamophobia in the Eu After 11 September 2001: Summary Report: Diane Pub. Co.

[58] Sheridan LP (2006) Islamophobia pre- and post-September 11th, 2001. J Interpers Violence 21: 317–336. 16443594 10.1177/0886260505282885

[59] Park J, Felix K, Lee G (2007) Implicit Attitudes Toward Arab-Muslims and the Moderating Effects of Social Information. Basic Appl Soc Psych 29: 35–45.

[60] Wang F, Yamagishi T (2005) Group-based trust and gender differences in China. Asian Journal of Social Psychology 8: 199–210.

[61] Ekehammar B, Akrami N, Araya T (2003) Gender differences in implicit prejudice. Pers Indiv Differ 34: 1509–1523.

[62] Fazio RH, Jackson JR, Dunton BC, Williams CJ (1995) Variability in automatic activation as an unobtrusive measure of racial attitudes: A bona fide pipeline? J Pers Soc Psychol 69: 1013–1027. 8531054 10.1037//0022-3514.69.6.1013

[63] Hofmann W, Gawronski B, Gschwendner T, Le H, Schmitt M (2005) A meta-analysis on the correlation between the implicit association test and explicit self-report measures. Pers Soc Psychol Bull 31: 1369–1385. 16143669 10.1177/0146167205275613

